# Harmonizing FDG PET quantification while maintaining optimal lesion detection: prospective multicentre validation in 517 oncology patients

**DOI:** 10.1007/s00259-015-3128-0

**Published:** 2015-07-30

**Authors:** Elske Quak, Pierre-Yves Le Roux, Michael S. Hofman, Philippe Robin, David Bourhis, Jason Callahan, David Binns, Cédric Desmonts, Pierre-Yves Salaun, Rodney J. Hicks, Nicolas Aide

**Affiliations:** Nuclear Medicine Department, François Baclesse Cancer Centre, Caen, France; Nuclear Medicine Department, University Hospital and EA3878 (GETBO) IFR 148, Brest, France; Centre for Molecular Imaging, Peter MacCallum Cancer Centre, East Melbourne and University of Melbourne, Melbourne, Australia; Nuclear Medicine Department, University Hospital, Avenue Côte de Nacre, 14000 Caen, France; INSERM 1199, François Baclesse Cancer Centre, Caen, France; Normandie University, Caen, France

**Keywords:** FDG PET/CT, Quantification, Harmonization, Standardized uptake value, Tumour imaging

## Abstract

**Purpose:**

Point-spread function (PSF) or PSF + time-of-flight (TOF) reconstruction may improve lesion detection in oncologic PET, but can alter quantitation resulting in variable standardized uptake values (SUVs) between different PET systems. This study aims to validate a proprietary software tool (EQ.PET) to harmonize SUVs across different PET systems independent of the reconstruction algorithm used.

**Methods:**

NEMA NU2 phantom data were used to calculate the appropriate filter for each PSF or PSF+TOF reconstruction from three different PET systems, in order to obtain EANM compliant recovery coefficients. PET data from 517 oncology patients were reconstructed with a PSF or PSF+TOF reconstruction for optimal tumour detection and an ordered subset expectation maximization (OSEM3D) reconstruction known to fulfil EANM guidelines. Post-reconstruction, the proprietary filter was applied to the PSF or PSF+TOF data (PSF_EQ_ or PSF+TOF_EQ_). SUVs for PSF or PSF+TOF and PSF_EQ_ or PSF+TOF_EQ_ were compared to SUVs for the OSEM3D reconstruction. The impact of potential confounders on the EQ.PET methodology including lesion and patient characteristics was studied, as was the adherence to imaging guidelines.

**Results:**

For the 1380 tumour lesions studied, Bland-Altman analysis showed a mean ratio between PSF or PSF+TOF and OSEM3D of 1.46 (95 %CI: 0.86–2.06) and 1.23 (95 %CI: 0.95–1.51) for SUV_max_ and SUV_peak_, respectively. Application of the proprietary filter improved these ratios to 1.02 (95 %CI: 0.88–1.16) and 1.04 (95 %CI: 0.92–1.17) for SUV_max_ and SUV_peak_, respectively. The influence of the different confounding factors studied (lesion size, location, radial offset and patient’s BMI) was less than 5 %. Adherence to the European Association of Nuclear Medicine (EANM) guidelines for tumour imaging was good.

**Conclusion:**

These data indicate that it is not necessary to sacrifice the superior lesion detection and image quality achieved by newer reconstruction techniques in the quest for harmonizing quantitative comparability between PET systems.

**Electronic supplementary material:**

The online version of this article (doi:10.1007/s00259-015-3128-0) contains supplementary material, which is available to authorized users.

## Introduction

Standardized uptake values (SUVs) extracted from FDG-PET/CT are being increasingly used as non-invasive quantitative imaging biomarkers in oncology. However, in order to validate SUV as a biomarker both in the local and multicentre setting, adequate reproducibility is required so that SUVs are comparable regardless of the imaging site or PET system used. By harmonizing both patient preparation as well as acquisition and reconstruction parameters, various groups, including the European Association Research Ltd (EARL) accreditation program [1], the North American Quantitative Imaging Biomarker Alliance (QIBA) [2] and Uniform Protocols in Clinical Trials (UPICT) [3, 4], aim to harmonize standardized uptake values (SUV) in the multicentre trial setting. Currently, centres running PET systems with advanced reconstruction algorithms are requested in some clinical trials to revert to older reconstruction so that their data is comparable to other centres with older systems. This issue was exemplified by the RATHL Lymphoma trial ([5]), which mandated that centres with point spread function (PSF) reconstruction or time of flight (TOF) disable these features when participating in the study. It is therefore important to develop strategies that enable use of newer reconstruction algorithms that improve lesion detection, whilst maintaining the compatibility of SUV with older systems.

While many sources of heterogeneity in SUV measurements can be overcome by complying with the European Association of Nuclear Medicine (EANM)/Society of Nuclear Medicine and Molecular Imaging (SNMMI) guidelines for PET tumour imaging [6–8], reconstruction-dependent variations require the use of an additional filtering step. It has been recently shown that it is possible to harmonize SUVs produced by an advanced reconstruction algorithm, such as PSF reconstruction, for example, to EANM standards by applying a filter during the reconstruction [9]. Unfortunately, this method requires the reconstruction of two data sets: one for optimal lesion detection and one for harmonized quantification, which is time consuming and requires supplementary digital storage.

To avoid the reconstruction of two data sets, a proprietary technical solution, marketed as EQ.PET (Siemens, Oxford, UK), has been developed to simultaneously allow optimal lesion detection and harmonized quantification from a single data set [10]. This software simultaneously presents the reconstruction that provides optimal lesion detection for diagnostic interpretation with harmonized SUV results.

This prospective multicentre trial sought to validate the ability of this proprietary solution to harmonize SUVs from oncological PET scans across different PET systems equipped either with PSF or PSF plus TOF reconstruction. As SUV is mainly used for therapy assessment, we mimicked a situation in which a patient would undergo pre-treatment and post-treatment scans on different generation PET systems by reconstructing the same raw PET data with an ordered subset expectation maximization (OSEM) algorithm known to meet EANM requirements, and a PSF or PSF plus TOF reconstruction designed for optimal tumour detection. A filter was then applied to the PSF or PSF plus TOF reconstruction to fulfil EANM requirements. We focused on SUV_max_ and SUV_peak_, the two most frequently used metrics in oncology, and included patients with non-small cell lung cancers, melanoma, non-Hodgkin lymphoma and liver metastases from colorectal cancer, thus covering all anatomical locations, tumours sizes and shapes against varying normal tissue backgrounds. The potential impact of several confounding factors [tumour size, organ location, lesion location in the field of view, patient body mass index (BMI)] on the accuracy of this methodology was investigated. As SUV reconstruction dependency is not the only source of variability, other technical and biological parameters, as well as compliance to EANM guidelines for PET tumour imaging [7], were also analysed.

## Materials and methods

### Patients

The PET exams of 517 consecutive patients from three centres referred for staging or restaging of non-small cell lung cancer (NSCLC), melanoma, non-Hodgkin lymphoma (NHL) or liver metastases from colorectal cancer were included over a 9-month period. Informed consent was waived for this type of study by the local ethics committee (Ref A12-D24-VOL13, *Comité de protection des personnes Nord-Ouest III*) since the scans were performed for clinical indications and the trial procedures were performed independent of usual clinical reporting.

### PET systems and cross-calibration of PET systems

Data from the following three PET systems were used for this study: a Biograph 6 TrueV with PSF reconstruction, a mCT with PSF+TOF, and a Biograph 64 TrueV with PSF reconstruction (Siemens Medical Solutions). Technical details regarding these systems can be found elsewhere [11, 12].

The daily calibration of each PET system was performed with a ^68^Ge source according to the integrated manufacturer’s protocol. The quarterly cross-calibration of each PET system was performed according to the EANM guidelines, as described elsewhere [6]. All clocks were synchronized weekly.

### Phantom preparation

The NEMA NU 2 anthropomorphic International Electrotechnical Commission body phantom set with six coaxial isocentred spheres with internal diameters of 10, 13, 17, 22, 28, and 37 mm (PTW Freiburg) was prepared according to the EANM guidelines with an ^18^F-FDG solution to achieve a sphere-to-background ratio of 10.

### Patient preparation

All patients were requested to fast for 6 h prior to injection of ^18^F-FDG. Upon arrival at each PET unit, patient height, weight and blood glucose level were recorded, and the body-mass index was calculated according to the following formula:

$$ BMI=\frac{weight(kg)}{heigh{t}^2\left({m}^2\right)}. $$

Patients were injected intravenously with ^18^F-FDG, followed by a planned 60 min rest in a warm room. The injected activity and the exact delay between injection and the start of the acquisition were recorded for each patient.

### PET/CT acquisition and reconstruction parameters (Table 1)

**Table 1:** PET/CT acquisition and reconstruction parameters for the three participating centres

Site and PET system		Centre 1 Biograph 6		Centre 2 Biograph mCT		Centre 3 Biograph 64			
PET acquisition	Duration per bed position	2 min 40 s (BMI ≤ 25) or 3 min 40 s (BMI > 25)		2 min 00 s		2 min 30 s (≤ 65 Kg), 3 min (65–85 Kg), 3 min 30 s (85–100 Kg), 4 min 00 s (> 100Kg)			
	Details	–	–	–	–		≤ 65 kg	65–100 Kg	> 100 Kg
	Reconstruction	OSEM3D	PSF	OSEM3D	PSF+TOF	OSEM3D	PSF	PSF	PSF
	Iterations/ Subsets	4i 8 s	3i 21 s	2i 24 s	2i 21 s	4i 8 s	3i 21 s	3i 21 s	3i 21 s
PET reconstruction	Post filter	5 mm	0 mm	4.4 mm	2 mm	3.5 mm	6 mm	5 mm	4 mm
	Matrix	168 × 168	168 × 168	200 × 200	200 × 200	168 × 168	168 × 168	168 × 168	168 × 168
	Pixel spacing	4.07 × 4.07	4.07 × 4.07	4.07 × 4.07	4.07 × 4.07	3.39 × 3.39	3.39 × 3.39	3.39 × 3.39	3.39 × 3.39
	Slice thickness	5 mm	5 mm	2.027 mm	2.027 mm	3 mm	3 mm	3 mm	3 mm
	EQ Filter	0 mm	6.9 mm	0 mm	6.3 mm	0 mm	2.4 mm	3.9 mm	4.9 mm
CT protocol	Voltage/intensity	120 kV/60mAs		120 kV/80mAs		140 kV/80mAs			
	Collimation/pitch	6*2 mm/ pitch 1		16*1.2 mm/ pitch 1		24*1.2 mm/ pitch 1			

Technical details for the CT acquisition of each PET/CT system can be found in Table 1. The administration of intravenous and/or oral CT contrast medium was recorded for each patient.

The PET acquisition was performed in 3D-mode with scatter and attenuation corrections. Patients were scanned from skull vertex or base to mid-thighs; the acquisition was extended to include the legs in melanoma patients with primary site of disease in the lower limbs.

All raw PET data were reconstructed with the local PSF or PSF+TOF settings designed to achieve optimal lesion detection. Raw PET data were also reconstructed with an OSEM-3D reconstruction algorithm known to fulfil the EANM guidelines.

### PET quantitative analysis

#### Phantom studies and calculation of the EQ.PET filter

For each reconstruction, 3D 50 % isocontour volumes of interest (VOIs) were drawn over the spheres and mean and maximum pixels values were recorded. The recovery coefficients (RCs) were calculated as the ratio between the measured and true activity concentration for each sphere.

For each PET system, the EQ.PET filter, formerly called the SUV_ref_ filter, was calculated on the phantom data of each PSF or PSF+TOF reconstruction as described in the paper by Kelly and Declerck [10]. Briefly, the voxel with the maximum activity for each sphere was used to calculate the RC. The RCs were then compared to a set of reference RCs to calculate the root mean square error (RMSE). This comparison was repeated while increasing the full width half maximum (FWHM) of the Gaussian kernel. The kernel size that minimized the RMSE compared to the reference RCs was then selected as the EQ.PET filter for that specific reconstruction and applied post-reconstruction. The reference RCs used were the optimal RCs published in the EANM procedure guidelines 1.0 for tumour imaging [6].

#### Patient studies

All PET exams were analysed on a prototype implementation of the EQ.PET functionality that is available for clinical use in Syngo.via (Siemens Medical Solutions). Displayed on the screen were the PSF or PSF+TOF reconstruction and the OSEM reconstruction. The EQ.PET filtered SUVs were calculated behind the scenes, without showing the filtered image. The appropriate EQ.PET filter for each PSF or PSF+TOF reconstruction was set as a default and displayed on the screen. On the PSF or PSF+TOF reconstruction, VOIs with a 50 % isocontour were drawn on a maximum of five lesions, including the hottest lesion per patient, with a maximum of two lesions per organ site. These VOIs were then automatically propagated on the OSEM reconstruction by re-computing a 50 % isocontour at the same location as in the PSF data set. SUV_peak_ was defined as a 1-cm^3^ sphere positioned within the lesion so as to maximise the enclosed average SUV. Background activity was measured in an automatically placed 3-cm diameter sphere in the right liver lobe and a 1-cm diameter and 2-cm height cylinder in the descending thoracic aorta.

With the current version of the EQ.PET software, the applied EQ.PET filter was not stored in the DICOM header, but all data, including screenshots of all measurements, were saved in a dedicated file.

For all lesions and backgrounds, the SUV_mean_, SUV_max_ and SUV_peak_, location and radial offset were recorded for PSF or PSF+TOF, PSF_EQ_ or PSF+TOF_EQ_ and OSEM3D. For this study, SUV_peak_ was not corrected for lean body mass. The short axis dimension of each lesion was measured on the axial CT slices when possible.

### Statistical analysis

For all sets of reconstructed data, RCs for all spheres were compared to EANM expected values by means of the root mean square error (RMSE) method. Details about the RMSE method can be found elsewhere [9]. The EQ Gaussian filter that minimises the RMSE when compared to EANM expected values was selected as the optimal filter for PSF or PSF+TOF reconstruction on each PET/CT system.

Quantitative data from clinical PET/CT examinations are presented as means (standard deviation, SD). The relationship between PSF or PSF+TOF, PSF_EQ_ or PSF+TOF_EQ_ and OSEM quantitative values was assessed with Bland-Altman plots [13]. The ratios between PSF_EQ_ or PSF+TOF_EQ_ and OSEM quantitative values (for SUV_max_, and SUV_peak_) according to lesion size, location, radial offset and patient’s BMI were compared using the Kruskal-Wallis test (with a post hoc Dunn test) for multiple groups comparison, or the Mann–Whitney test for unpaired samples when appropriate. For lesion size, the ratios between PSF_EQ_ or PSF+TOF_EQ_ and OSEM SUVs were dichotomized in three groups (< 1, 1–2 and > 2 cm). For radial offset, an arbitrary cutoff of 75 mm was used to discriminate between centrally located and peripherally located lesions within the field of view (FOV) [10]. For all tests, a two-tailed *p* value of less than 0.05 was considered statistically significant. Graphs and analyses were carried out using Prism (GraphPad software, La Jolla, CA).

## Results

### Phantom studies

**Fig. 1:**
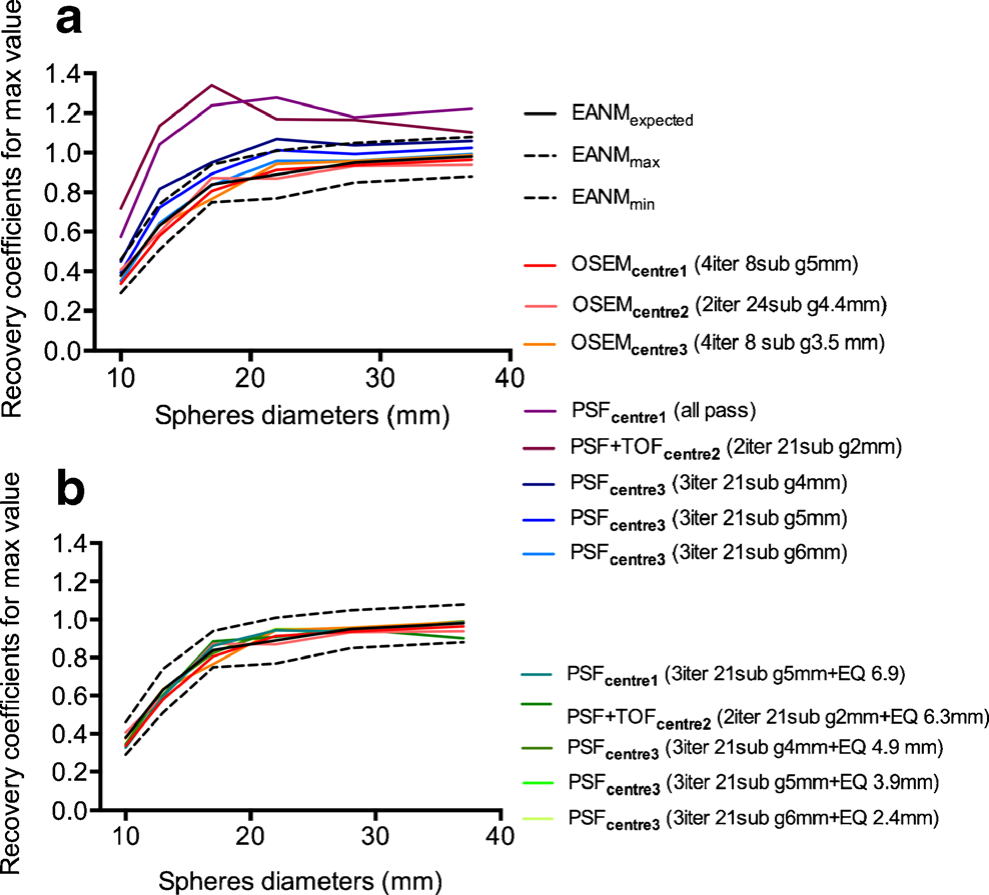
Recovery coefficients extracted from NEMA NU2 phantom acquisition for maximum values for (**a**) PSF or PSF+TOF and OSEM3D reconstructions, and (**b**) for PSF_EQ_ or PSF+TOF_EQ_ and OSEM3D

Figure 1 shows the recovery coefficients for maximum values for all PSF or PSF+TOF, PSF_EQ_ or PSF+TOF_EQ_ and OSEM3D reconstructions. The curves for almost all PSF or PSF+TOF reconstructions were found to be above the EANM expected values, except for Centre 3, using amongst others a PSF reconstruction with a 6-mm Gaussian filter. For the larger spheres, most PSF or PSF+TOF RCs were even greater than 1, which is probably due to the Gibbs artefact in PSF modelling [14–16]. The OSEM3D recovery curves were all closely matched with EANM expected values. After application of the EQ.PET filter (see Table 1), all recovery curves aligned within the EANM expected curve.

### Patient studies

#### Compliance to guidelines for tumour imaging

Over a 9-month period, three consecutive cross-calibration procedures were performed in each centre and were found to comply with the EANM guidelines: cross calibrations factors were 1.01, 0.99 and 1.00 for centre 1, 1.07, 1.06 and 1.06 for centre 2, and 1.02, 0.98 and 0.97 for centre 3. All clocks were synchronized weekly and were never out of synchronization more than 2 min. The mean (SD) administered ^18^F-FDG doses for centre 1 was 3.99 (0.18) MBq/kg, for centre 2 3.97 (0.25) MBq/kg, and for centre 3 3.58 (0.21) MBq/kg. The mean (SD) delay between the administration of ^18^F-FDG and the start of the PET acquisition was 63 (6.02) minutes for centre 1, 63 (5.59) minutes for centre 2, and 73 (13.04) minutes for centre 3. Overall, the EANM 2.0 guidelines for PET tumour imaging were fulfilled in 469/517 patients (91 %). At the time of injection, the mean (SD) blood glucose level was 5.86 (2.36) mmol/l.

#### Validation of the EQ software to overcome reconstruction-dependent variability

The characteristics of the 517 patients can be found in Table 2. In total, 1380 tumour lesions were analyzed, of which 1167 had a measurable diameter on low-dose CT. Intravenous contrast was administered in 104 cases. No oral contrast medium was used.

**Table 2:** Patient characteristics

Characteristic (*n* = 517)	
Sex ratio male/female	2.1
Age (years), mean (SD)	64 (11)
BMI (kg/m^2^), mean (SD)	26 (5)
Glycemia (mmol/l), mean (SD)	5.9 (2.4)
Histological diagnosis, *n* (%)	
Colorectal cancer	79 (15)
Adenocarcinoma,	73 (92)
N/A	6 (8)
Mean number of lesions per patient	2.73
Melanoma	59 (11)
Mean number of lesions per patient	2.34
Non-Hodgkin lymphoma	121 (23)
DLBCL,	58 (48)
FL	34 (28)
Other	27 (22)
N/A	2 (2)
Mean number of lesions per patient	2.70
Non-small cell lung cancer	258 (50)
Adenocarcinoma,	161 (62)
Squamous cell carcinoma	78 (30)
Other	10 (4)
N/A	9 (4)
Mean number of lesions per patient	2.71

*BMI* body mass index, *N/A* not available, *DLBCL* diffuse large B cell lymphoma, *FL* follicular lymphoma

**Fig. 2:**
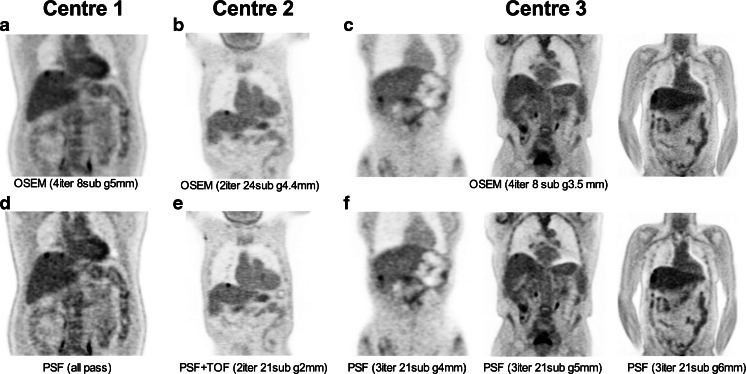
Representative images of OSEM (**a**, **b**, **c**) and PSF or PSF+TOF (**d**, **e**, **f**) reconstructions for the three participating centres. All images have been scaled on the same maximum value (SUV = 5). All selected patients are patients with liver metastases from colorectal cancer. Note the increase in apparent tracer uptake in PSF or PSF+TOF images as compared to a conventional OSEM algorithm fulfilling the EANM requirements. This increase was particularly present in the PSF or PSF+TOF images without filter or when applying a Gaussian filter with a small kernel (centres 1 and 2)

Representative images for each OSEM and PSF or PSF+TOF reconstructions are illustrated in Fig. 2.

**Fig. 3:**
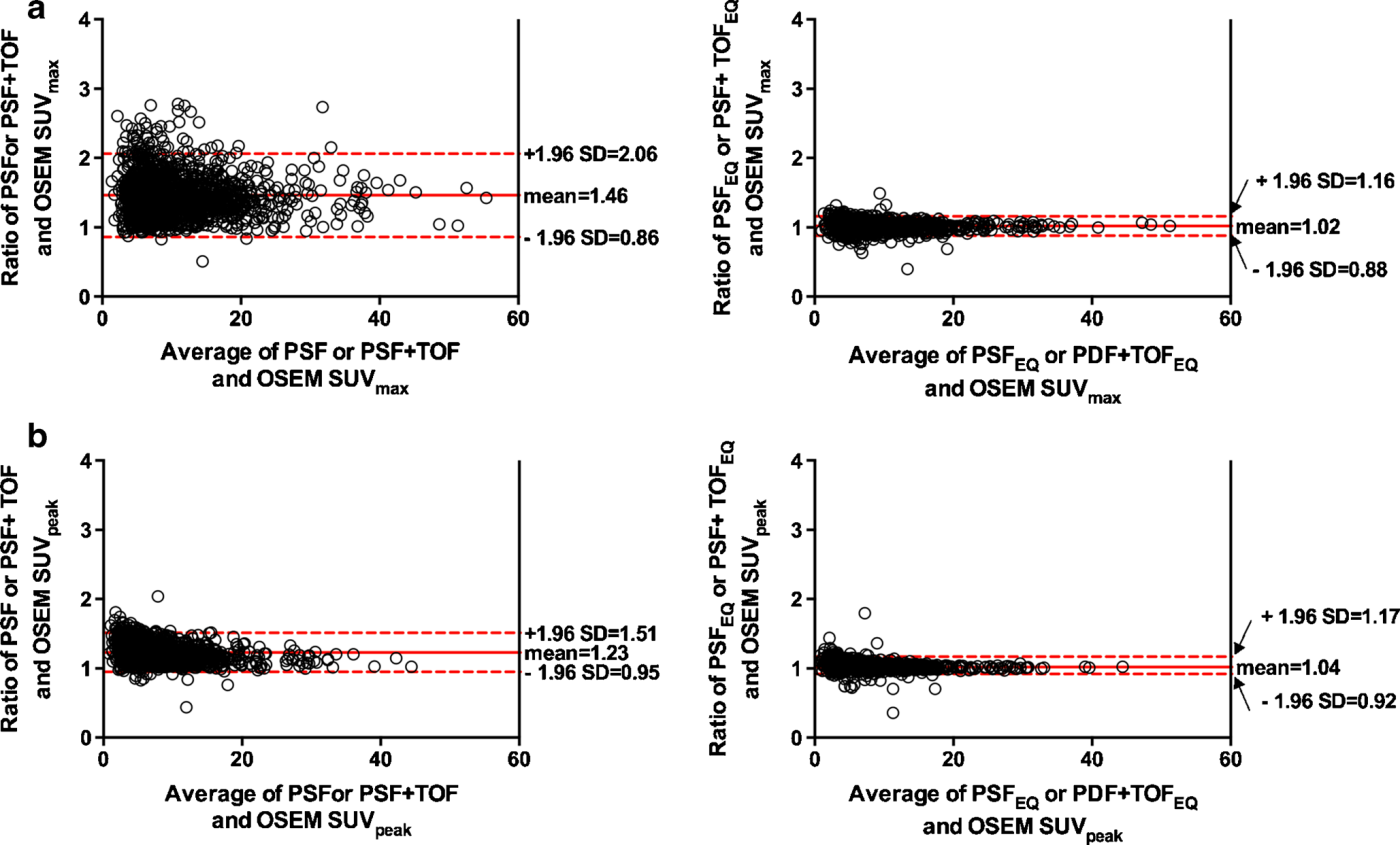
Relationship between quantitative values extracted from PSF/PSF+TOF or PSF_EQ_/PSF+TOF_EQ_ and OSEM images, assessed using Bland-Altman plots for SUV_max_ (**a**) and SUV_peak_ (**b**) in tumour lesions

For tumours, the mean SUV_max_ (SD) for OSEM, PSF or PSF+TOF and PSF_EQ_ or PSF+TOF_EQ_ reconstructions was 9.3 (6.1), 13.2 (8.2) and 9.4 (6.1), respectively. The mean SUV_peak_ (SD) for OSEM, PSF or PSF+TOF and PSF_EQ_ or PSF+TOF_EQ_ reconstructions was 7.4 (5.3), 8.9 (5.8) and 7.6 (5.3), respectively. The mean ratios between PSF or PSF+TOF and OSEM3D reconstructions for SUV_max_ and SUV_peak_ were 1.46 (95 %CI: 0.86–2.06) and 1.23 (95 %CI: 0.95–1.51), respectively (Fig. 2). After application of the EQ.PET filter, this was reduced to 1.02 (95 %CI: 0.88–1.16) and 1.04 (95 %CI: 0.92–1.17) respectively (Fig. 3).

**Fig. 4:**
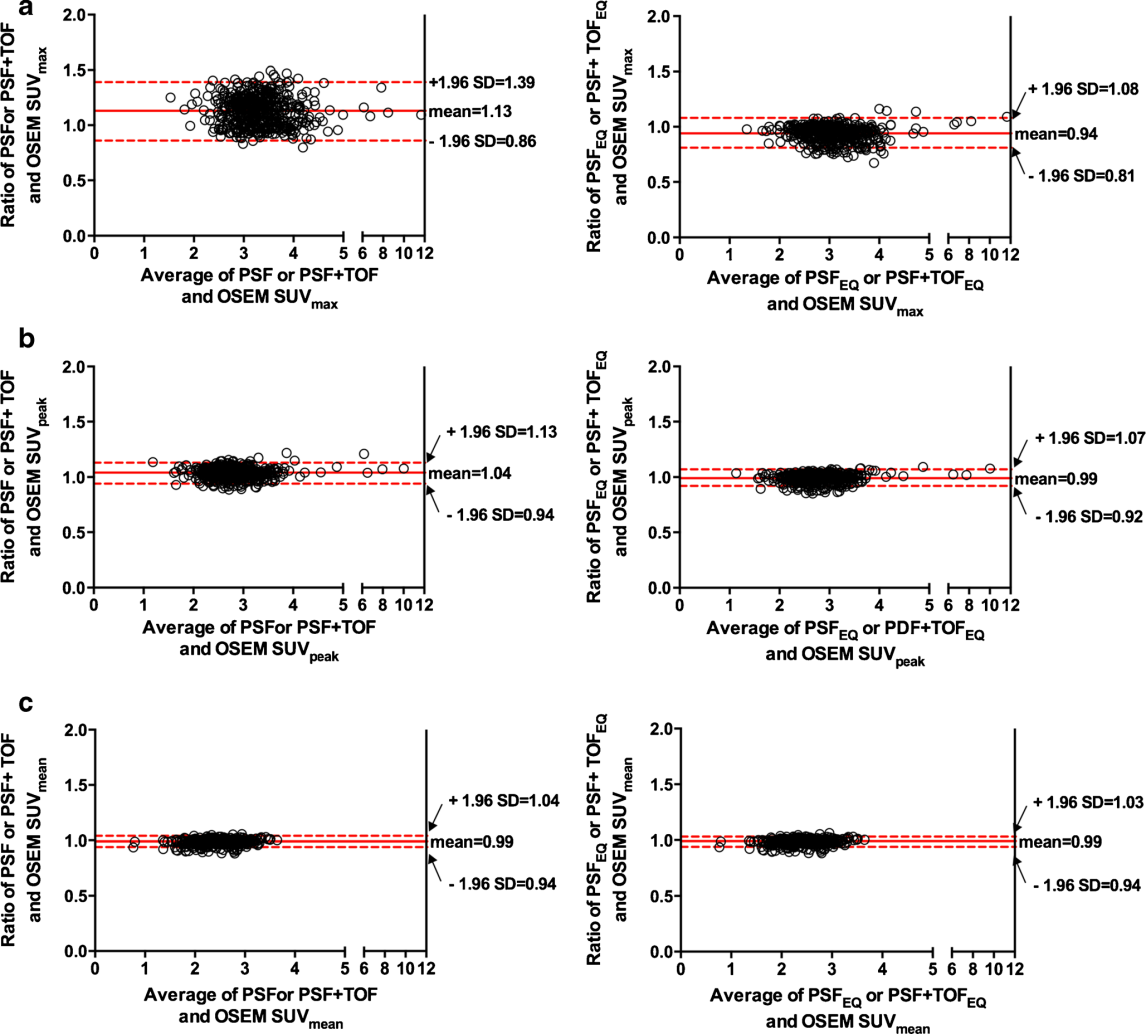
Relationship between quantitative values extracted from PSF/PSF+TOF or PSF_EQ_/PSF+TOF_EQ_ and OSEM images, assessed using Bland-Altman plots for SUV_max,_ (**a**) SUV_peak_ (**b**) and SUV_mean_ in the liver background (**c**)

For the liver background, the mean SUV_max_ (SD) for OSEM, PSF or PSF+TOF and PSF_EQ_ or PSF+TOF_EQ_ reconstructions were 3.2 (0.7), 3.5 (0.8) and 3.0 (0.7), respectively. The mean SUV_peak_ (SD) for OSEM, PSF or PSF+TOF and PSF_EQ_ or PSF+TOF_EQ_ reconstructions were 2.8 (0.6), 2.9 (0.7) and 2.8 (0.7), respectively. The mean ratios between PSF or PSF+TOF and OSEM3D for SUV_max,_ SUV_peak_ and SUV_mean_ were 1.13 (95 %CI: 0.86–1.39), 1.04 (95 %CI: 0.94–1.13) and 0.99 (95 %CI: 0.94–1.04) respectively (Fig. 4), and 0.94 (95 %CI: 0.81–1.08), 0.99 (95 %CI: 0.92–1.07) and 0.99 (95 %CI: 0.94–1.03), respectively, after application of the EQ.PET filter. The results for the mediastinal background can be found in the supplemental data (supplemental Fig. 1).

**Fig. 5:**
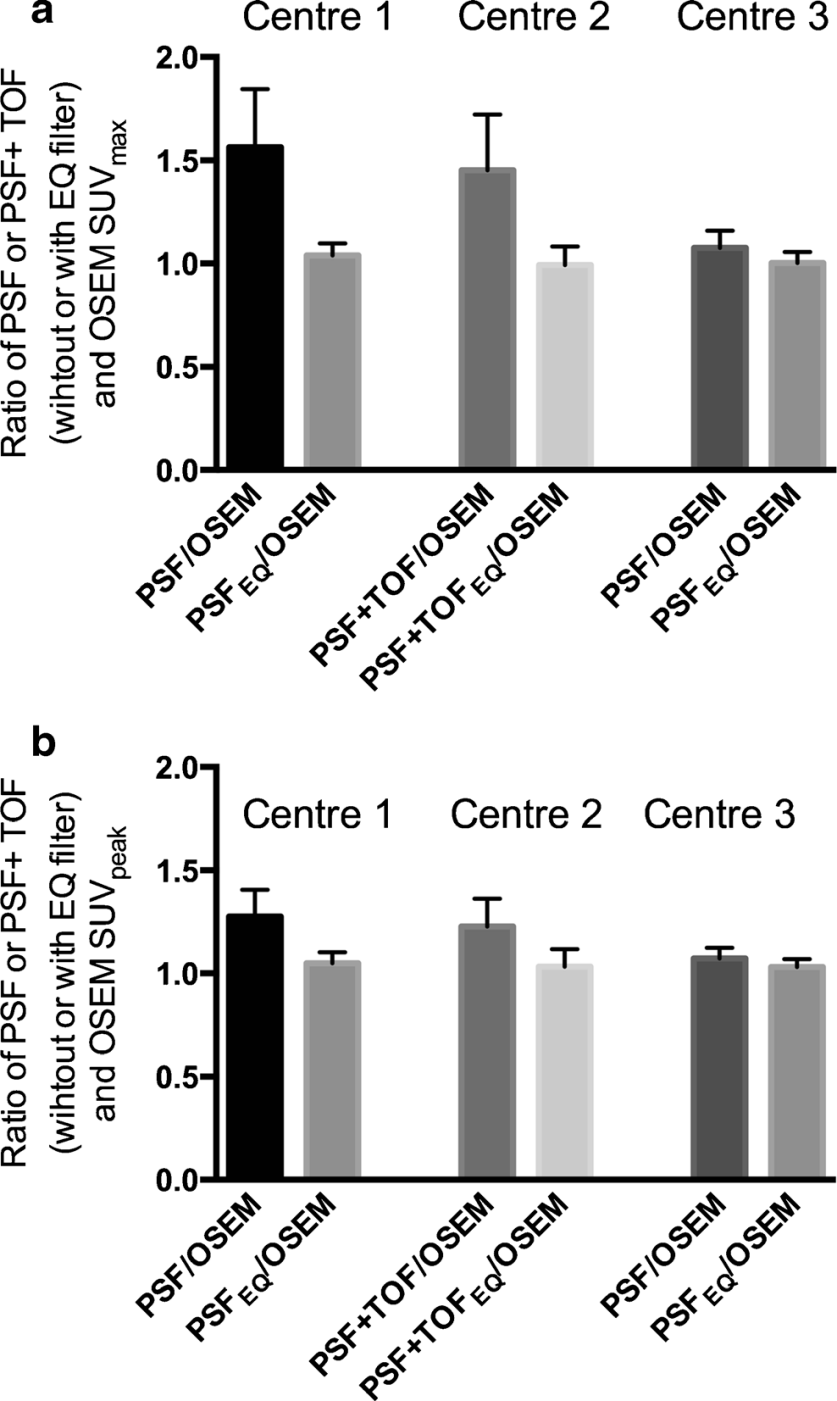
Relative impact of the PET technology on quantitative values. Mean (SD) ratio of SUV_max_ (**a**) and SUV_peak_ (**b**) obtained with a conventional OSEM algorithm and those obtained with PSF or PSF+TOF reconstructions are shown before and after application of the EQ technology. Data are shown for the three participating centres. Reconstruction settings and EQ filter values are detailed in Table 1. Note the difference in ratio between PSF and OSEM data in centres 1 and 3 using the same PET system (PSF reconstruction), either with no post filtering (centre 1) or with a Gaussian filter (kernel ranging from 4 to 6 mm, centre 3)

For the different centres, mean (SD) ratio between SUV_max_ obtained with a conventional OSEM algorithm and those obtained with PSF reconstructions in centres 1 and 3 were 1.57 (0.28) and 1.08 (0.08) before and 1.04 (0.06) and 1.00 (0.06) after application of the EQ technology, respectively. For PSF+TOF reconstruction of centre 2, the ratio was 1.45 (0.27) before and 0.99 (0.09) after application of the EQ technology. Mean (SD) ratio between SUV_peak_ obtained with a conventional OSEM algorithm and those obtained with PSF reconstructions in centres 1 and 3 were 1.28 (0.18) and 1.07 (0.05) before and 1.05 (0.05) and 1.03 (0.04) after application of the EQ technology, respectively. For PSF+TOF reconstruction of centre 2, the ratio was 1.23 (0.13) before and 1.03 (0.09) after application of the EQ technology (Fig. 5).

**Fig. 6:**
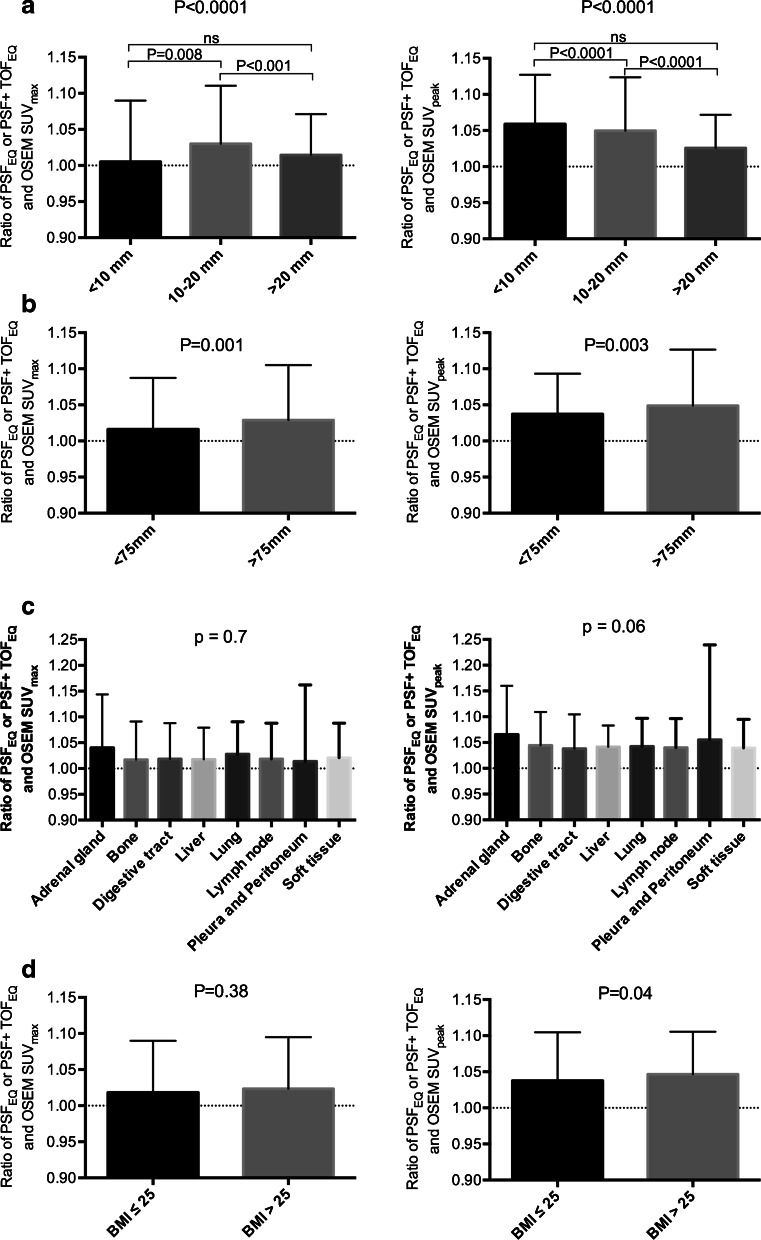
Impact of the size of the lesion (**a**), the location of the lesion across the field of view [radial offset (**b**)], the anatomical site of the lesion (**c**), and patient BMI (**d**) on the ratio between PSF_EQ_/PSF+TOF_EQ_ and OSEM PET quantitative values (*left* panels SUV_max_, *right* panels SUV_peak_). Note that 213 lesions were not measurable and therefore not included in the “per size” analysis. The differences among different groups were tested with the Kruskal-Wallis test, and a post hoc test was performed with the Dunn test for multiple comparisons; *, **, and *** indicate two-tailed *p* < 0.05, *p* < 0.01, and *p* < 0.001, respectively

The impact of potential confounders on the ratios between PSF_EQ_ or PSF+TOF_EQ_ and OSEM3D reconstructions for SUV_max,_ and SUV_peak_ are shown in Fig. 6. All ratios were found to be within the 1.05 limit, except for a 1.07 ratio for the location adrenal gland for SUV_peak_. For all confounding factors, ratios for SUV_peak_ were found to be slightly higher than for SUV_max_. SUV_peak_ ratios varied from 1.02 to 1.07 versus 1.01 to 1.04 for SUV_max_.

Regarding lesion short axis, ratios for SUV_peak_ were higher for smaller lesions, compared to larger lesions. This effect was not seen for SUV_max_. Regarding radial offset, ratios tended to be lower close to the centre of the field of view for both SUV_max_ and SUV_peak_. Regarding body location, no differences were found. Lastly, ratios for SUV_peak_ for patients with a BMI > 25 tended to be slightly higher than for normal weight patients.

Amongst the 1380 analysed lesions, Bland-Altman plots identified 46 outliers for the SUV_max_ and SUV_peak_ values, for which the ratios of SUV_max_ or SUV_peak_ PSF_EQ_ or PSF+TOF_EQ_ and SUV_max_ or SUV_peak_ OSEM were outside the limits of the confidence intervals. The majority of these outliers corresponded to lesions with a short axis diameter ≤ 2 cm (63 %), and the majority of outliers were lung and nodal lesions (63 %).

## Discussion

This prospective multicentre study in tumour PET imaging demonstrates that it is possible to perform optimal lesion detection while achieving harmonized quantification from a single PET acquisition and processed data set. This enables PET centres running new generation PET systems with advanced reconstruction protocols to benefit from optimal image quality, whilst not compromising quantitative capability.

The validation of a proprietary software solution, EQ.PET, was performed on PET data from three different PET systems equipped with advanced reconstruction algorithms in a large series of patients with different cancer types. It is noteworthy that two centres used the same PSF algorithm but applied a different post filter, highlighting the need to harmonize quantitative values even in centres running similar equipment. This finding is in concordance with recent results from the Clinical Trials Network of the SNMMI [17] and is clearly illustrated in Fig. 5, where ratios between SUVs obtained with a conventional OSEM algorithm and those obtained with PSF reconstruction are substantially higher in centre 1 using PSF modelling without post filtering, as compared to centre 3 using PSF modelling with a smoothing filter. This issue of variation of acquisition/reconstruction settings and thus system performance, even in centres running the same PET system, was also recently reported in a national survey of PET/CT operations in Austria [18].

As variability in SUV is not only reconstruction dependent, we analysed the biological and technical factors, and the adherence to the EANM guidelines. Compliance to these guidelines including clock synchronization, cross-calibration between PET systems and dose calibrator, injected dose and delay between injection and imaging was found to be good in this study of consecutive patients scanned in routine clinical practice. Despite this good compliance, we found mean ratios between PSF or PSF+TOF and OSEM3D reconstructions for SUV_max_ and SUV_peak_ equal to 1.46 and 1.23, respectively. This may impact therapy assessment either with the EORTC or PERCIST criteria.

Our results demonstrate, by mimicking a situation in which a patient would undergo the pre-therapy and post-therapy PET scans on different generation PET systems, that it is possible to minimise reconstruction dependent variability. We found that with EQ.PET filter for each different PSF or PSF+TOF reconstruction, we were able to harmonize PET quantitative data for tumours to achieve a mean ratio of 1.02 for SUV_max_ and 1.04 for SUV_peak_, with narrow confidence interval in both cases.

The influence of the different confounding factors studied, including lesion size, radial offset, location and BMI, was found to be less than 5 % on average. However, these differences are probably not clinically significant as they were well below the threshold to discriminate between responders and non-responders for therapy assessment either with the EORTC (25 %) or PERCIST (30 %) criteria [19, 20]. Of note, ratios were significantly higher in lateral lesions as opposed to centrally located lesions. This is explained by a more pronounced effect of PSF reconstruction at the edges of the FOV, and that a similar EQ filter was used irrespective of the location of the lesion. Results regarding ratios of SUV_peak_ in lesions smaller than the ROI used for computation of this metric (all lesions in the < 1 cm group and a part of the lesions in the 1–2 cm group) can be explained by a more pronounced partial volume effect within the ROI in OSEM images as compared to PSF images in these small lesions, and that a similar EQ filter was used irrespective of the size of the lesion. As stated in the PERCIST paper by Wahl et al. [20], lesions should at least be 2 cm for accurate SUVpeak measurements, and smaller lesions should show sufficient FDG uptake. No explanation was found for the higher ratios in overweight patients as compared to normal weight patients, a finding in disagreement with studies showing that SUVpeak is less sensitive than SUV_max_ to image noise [21]. Again, these differences are unlikely to be clinically significant.

The impact of advanced algorithms on PET quantitation in liver, an organ frequently used as reference background for FDG PET imaging, was found to be minimal, as compared to tumour lesions. This finding is significant because it suggests that using a tumour-to-liver ratio is insufficient to correct for reconstruction variability. It strengthens the need to harmonize SUVs not only when it is taken alone, but also when using tumour-to-liver SUV ratios, such as in the Deauville criteria for interim and end-treatment PET scans in lymphoma patients [22–24].

For this study the current EANM expected values were set as the reference standard. However, the EQ.PET filter could be adapted to meet any given standard. This is important in the context of evolving guidelines. For example, while first version the EANM guidelines for tumour imaging recommend using reconstruction parameters providing a RC value ranging from 0.88 to 1.08 for the 37 mm sphere [6], the EARL accreditation program evolved to a recommended RC value ranging from 0.95 to 1.16 [7].

To our knowledge, this is the first study testing an integrated software solution for harmonized quantification. The problem of substantially higher SUVs (up to 66 %) for new generation PET systems with advanced reconstruction algorithms as compared to conventional OSEM algorithms has been signalled by several authors [25–30]. In a previous prospective single centre study in NSCLC patients, collaborators in this trial showed that by applying a filter to the PSF reconstruction, it was possible to harmonize SUVs to an OSEM3D reconstruction known to adhere to the EANM standards. However, the reconstruction of two data sets remained mandatory. The advantages of EQ.PET compared to our previously proposed methodology are the reconstruction of just one data set, and the possibility to apply EQ.PET on retrospective series of PET scans as the EQ.PET filter is applied post-reconstruction. Also, as pointed out by Boellaard [31], patients are frequently included in clinical trials after the first PET examination has been performed, requiring a new PET examination to be performed in accordance with the trial recommendations. The EQ technology could be used in this situation.

A limitation of this study is that this is a software solution developed for, and applied only to, scanners and reconstruction algorithms of the company that developed this product. Further research is needed to assess whether the EQ filter can be applied to other PET systems to facilitate a vendor-neutral software solution, or whether similar non-proprietary solutions can achieve similar results independent of scanner type or manufacturer. Other limitations are that with the current test version of EQ.PET, filtered data cannot be exported as DICOM files, and EQ.PET filtered data can’t be visually inspected.

## Conclusion

These data indicate that it is not necessary to sacrifice the superior lesion contrast and image quality achieved by newer reconstruction techniques in the quest for harmonizing quantitative comparability between PET systems. This is particularly applicable to multi-centre trials, and may provide the opportunity to provide accurate quantification for restaging even when the patient is scanned on a different device.

## Electronic supplementary material

Supplemental Figure 1 Relationship between quantitative values extracted from PSF/PSF+TOF or PSF_EQ_/PSF+TOF_EQ_ and OSEM images, assessed using Bland-Altman plots for SUV_max,_ SUV_peak_ and SUV_mean_ in the mediastinal background_._ (GIF 80 kb)

High Resolution Image (TIFF 11691 kb)
