# Supplementary figures and images for: Harmonizing FDG PET quantification while maintaining optimal lesion detection: prospective multicentre validation in 517 oncology patients
Source: Eur J Nucl Med Mol Imaging. 2015 Jul 30;42(13):2072–82. doi: 10.1007/s00259-015-3128-0 (PMC4623085; doi:10.1007/s00259-015-3128-0)

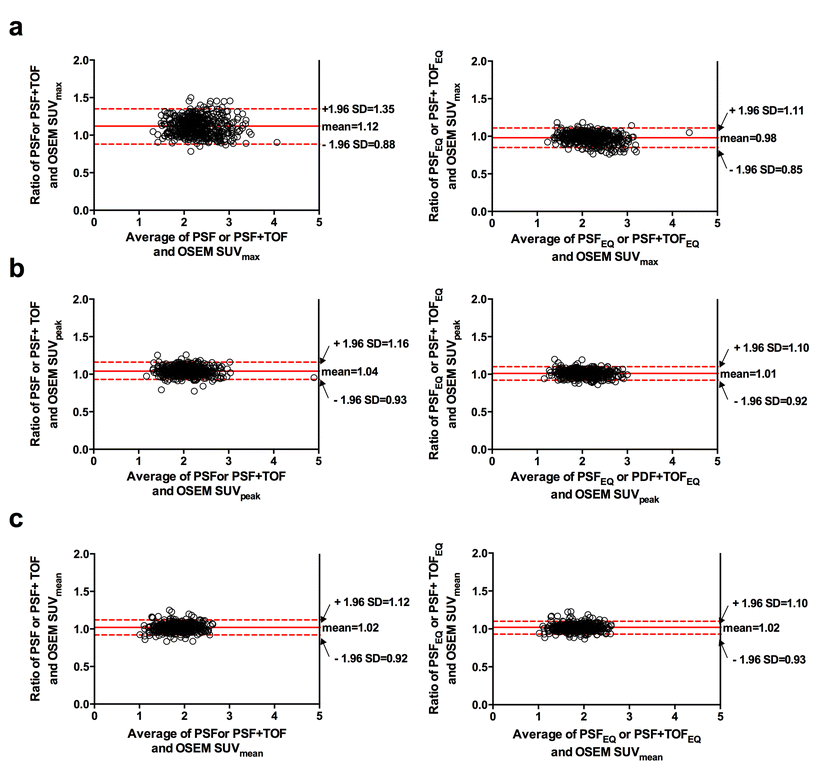

Supplement: Supplementary file 1 — Relationship between quantitative values extracted from PSF/PSF+TOF or PSFEQ/PSF+TOFEQ and OSEM images, assessed using Bland-Altman plots for SUVmax, SUVpeak and SUVmean in the mediastinal background. (GIF 80 kb) [file 259_2015_3128_Fig7_ESM.gif]

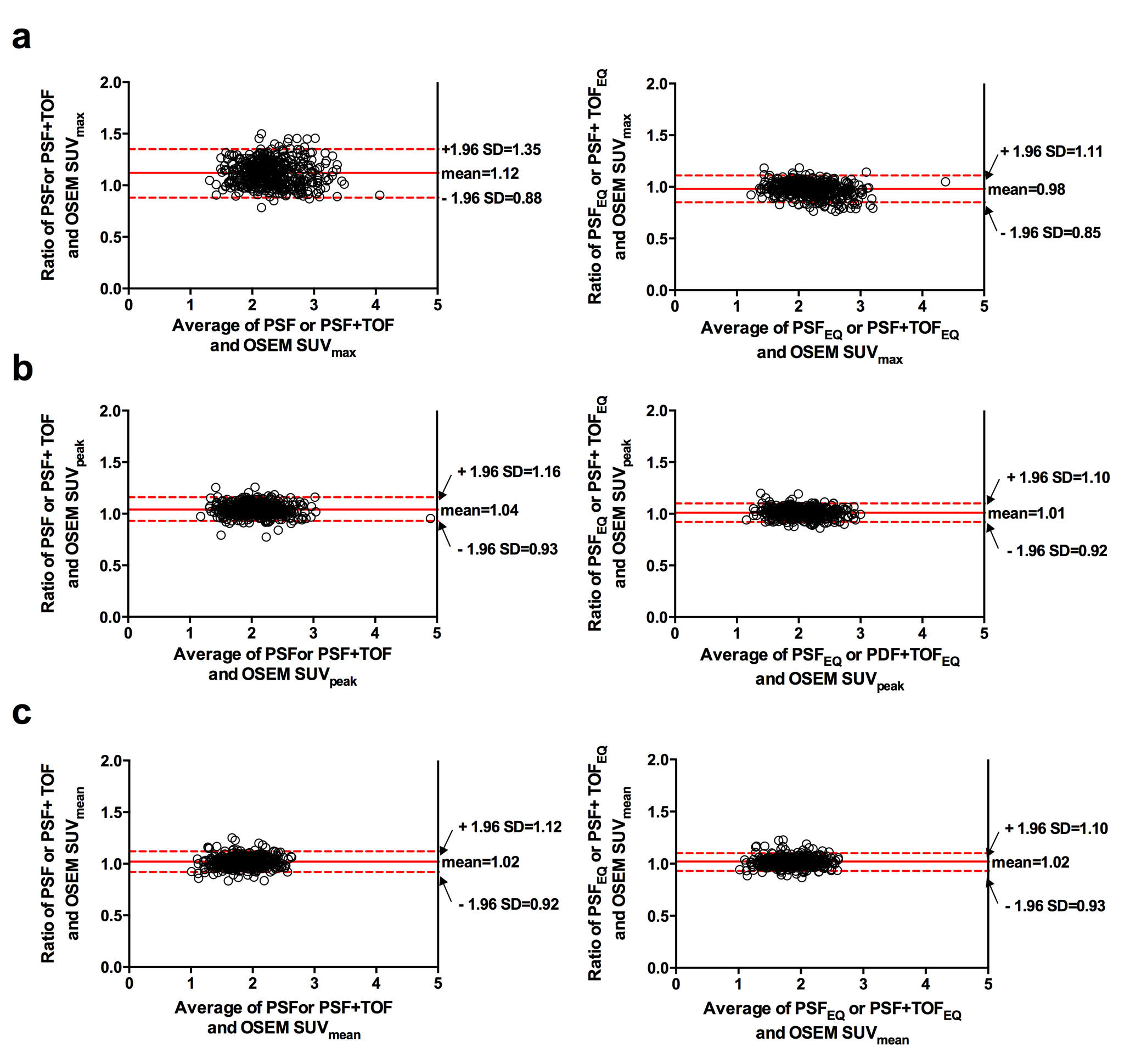

Supplement: Supplementary file 2 — High Resolution Image (TIFF 11691 kb) [file 259_2015_3128_MOESM1_ESM.tiff]
